# Supplementary material for: Prevention and Management of Iron Deficiency/Iron-Deficiency Anemia in Women: An Asian Expert Consensus
Source: Nutrients. 2023 Jul 13;15(14):3125. doi: 10.3390/nu15143125 (PMC10383547; doi:10.3390/nu15143125)

Table S1. Delphi results for statements relating to the identification of women at risk for ID/IDA.

Statements on identification of ID/IDA		Clarified statement	Round 1 rating	Round 2 rating	Result	Round 2 grading
1.1.	ID in women of reproductive age primarily results from an imbalance between iron intake and physiologic blood loss through menstrual bleeding or previous pregnancy.		88% (Retained)		Retained	B
1.2.	IDA develops when iron intake is insufficient to meet the iron demand of erythropoiesis.		100% (Retained)		Retained	B
1.3.	IDA typically manifests late in the clinical progression of iron-deficiency erythropoiesis.		100% (Retained)		Retained	U
1.4.	Pregnant women are at high risk for ID and the development of IDA.		100% (Retained)		Retained	U
1.5.	Non-pregnant adult women are at high risk for ID and the development of IDA.		75% (Included in Round 2)	44% (Non-consensus)	No consensus	D
1.6.	Adolescent girls are at high risk for ID and the development of IDA.		88% (Retained)		Retained	B
1.7.	Perimenopausal women are at high risk for ID and the development of IDA.		63% (Included in Round 2)	44% (Non-consensus)	No consensus	D
1.8.	Early detection of ID in pregnant women can help prevent progression to IDA as well as adverse consequences of ID without anemia.		100% (Retained)		Retained	U
1.9.	Early detection of ID in non-pregnant adult women can help prevent progression to IDA as well as adverse consequences of ID without anemia.		100% (Retained)		Retained	U
1.10.	Early detection of ID in adolescent girls can help prevent progression to IDA as well as adverse consequences of ID without anemia.		100% (Retained)		Retained	U
1.11.	Early detection of ID in perimenopausal women can help prevent progression to IDA as well as adverse consequences of ID without anemia.		88% (Retained)		Retained	B
1.12.	Pregnancy in multiparous women should prompt assessment of anemia and iron status to detect ID/IDA.		100% (Retained)		Retained	U
1.13.	Pregnant women with heavy uterine bleeding should prompt assessment of anemia and iron status to detect ID/IDA.		100% (Retained)		Retained	U
1.14.	Pregnant women who are not taking an iron supplement should prompt assessment of anemia and iron status to detect ID/IDA.		88% (Retained)		Retained	B
1.15.	Postpartum women with blood loss greater than 500 mL after delivery should prompt assessment of anemia and		100% (Retained)		Retained	U

Statements on identification of ID/IDA		Clarified statement	Round 1 rating	Round 2 rating	Result	Round 2 grading
	iron status to detect ID/IDA.					
1.16.	Postpartum women with uncorrected anemia detected in the antenatal period should prompt assessment of anemia and iron status to detect ID/IDA.		100% (Retained)		Retained	U
1.17.	Postpartum women with symptoms suggestive of anemia after delivery should prompt assessment of anemia and iron status to detect ID/IDA.		100% (Retained)		Retained	U
1.18.	Non-pregnant adult women with heavy menstrual bleeding should prompt assessment of anemia and iron status to detect ID/IDA.		100% (Retained)		Retained	U
1.19.	Adolescent girls with heavy menstrual bleeding should prompt assessment of anemia and iron status to detect ID/IDA.		100% (Retained)		Retained	U
1.20.	Adolescent girls experiencing fatigue should prompt assessment of anemia and iron status to detect ID/IDA.		100% (Retained)		Retained	U
1.21.	Adolescent girls with decreased verbal learning and memory should prompt assessment of anemia and iron status to detect ID/IDA.		88% (Retained)		Retained	B
1.22.	Perimenopausal women with heavy menstrual bleeding should prompt assessment of anemia and iron status to detect ID/IDA.		100% (Retained)		Retained	U
1.23.	Perimenopausal women experiencing fatigue should prompt assessment of anemia and iron status to detect ID/IDA.		100% (Retained)		Retained	U
1.24.	Perimenopausal women experiencing impaired exercise capacity should prompt assessment of anemia and iron status to detect ID/IDA.		100% (Retained)		Retained	U
1.25.	Pregnant women with pallor should prompt assessment of anemia and iron status to detect ID/IDA.		New statement (Included in Round 2)	78% (Non-consensus)	No consensus	B
1.26.	Postpartum women with pallor should prompt assessment of anemia and iron status to detect ID/IDA.		New statement (Included in Round 2)	89% (Retained)	Retained	B
1.27.	Adolescent girls with pallor should prompt assessment of anemia and iron status to detect ID/IDA.		New statement (Included in Round 2)	89% (Retained)	Retained	B
1.28.	Perimenopausal women with pallor should prompt assessment of anemia and iron status to detect ID/IDA.		New statement (Included in Round 2)	89% (Retained)	Retained	B
1.29.	Non-pregnant adult women with pallor should prompt assessment of anemia and iron status to detect ID/IDA.		New statement (Included in Round 2)	100% (Retained)	Retained	U
				Retained (n)	26	

Table S2. Delphi results for statements relating to the diagnosis and assessment of ID/IDA in women.

Statements on diagnosis and assessment of ID/IDA		Clarified statement	Round 1 rating	Round 2 rating	Result	Round 2 grading
2.1.	Serum ferritin below the normal range is the most specific early marker for the diagnosis of ID in otherwise healthy women.		88% (Retained)		Retained	B
2.2.	Serum ferritin is the most effective single test for the diagnosis of ID in pregnancy.		75% (Included in Round 2)	75% (Non-consensus)	No consensus	C
2.3.	Normal or borderline serum ferritin does not necessarily exclude ID.		63% (Included in Round 2)	88% (Retained)	Retained	B
2.4.	In cases with borderline serum ferritin, a more comprehensive iron panel comprising serum iron, total iron-binding capacity (TIBC), and transferrin saturation (TSAT) should be used to confirm ID.		75% (Included in Round 2)	100% (Retained)	Retained	U
2.5.	Serum hepcidin measurement is not adequately standardized for routine diagnosis of ID.		63% (Included in Round 2)	88% (Retained)	Retained	B
2.6.	A serum ferritin threshold of <15 µg/L is diagnostic of ID in otherwise healthy pregnant women in their first trimester of pregnancy.		75% (Included in Round 2)	50% (Non-consensus)	No consensus	D
2.7.	A serum ferritin threshold of <15 µg/L is diagnostic of ID in otherwise healthy non-pregnant adult women.		100% (Retained)		Retained	U
2.8.	A serum ferritin threshold of <15 µg/L is diagnostic of ID in otherwise healthy adolescent girls.		100% (Retained)		Retained	U
2.9.	A serum ferritin threshold of <15 µg/L is diagnostic of ID in otherwise healthy perimenopausal women.		100% (Retained)		Retained	U
2.10.	A serum ferritin threshold of <70 µg/L is diagnostic of ID in non-pregnant adult women with infection or inflammation.		100% (Retained)		Retained	U
2.11.	A serum ferritin threshold of <70 µg/L is diagnostic of ID in adolescent girls with infection or inflammation.		100% (Retained)		Retained	U
2.12.	A serum ferritin threshold of <70 µg/L is diagnostic of ID in perimenopausal women with infection or inflammation.		100% (Retained)		Retained	U
2.13.	A normal CRP alone should be used to exclude elevated serum ferritin caused by the acute-phase response to infection or inflammation.	Routinely available serum inflammatory markers may be used to exclude elevated serum ferritin caused by the acute-phase response to infection or inflammation.	63% (Included in Round 2)	75% (Non-consensus)	No consensus	C

Statements on diagnosis and assessment of ID/IDA		Clarified statement	Round 1 rating	Round 2 rating	Result	Round 2 grading
2.14.	A normal AGP alone should be used to exclude elevated serum ferritin caused by the acute-phase response to infection or inflammation.		25% (Excluded from Round 2)		Removed	
2.15.	A combination of normal CRP and AGP should be used to exclude elevated ferritin caused by the acute-phase response to infection or inflammation.		38% (Excluded from Round 2)		Removed	
2.16.	IDA is a composite diagnosis based on Hb concentration and serum ferritin.	IDA can be readily diagnosed by assessing Hb and confirmed by testing serum ferritin.	75% (Included in Round 2)	88% (Retained)	Retained	B
2.17.	Measurement of Hb concentration alone cannot be reliably used to establish a diagnosis of ID.		75% (Included in Round 2)	75% (Non-consensus)	No consensus	C
2.18.	Routine Hb measurement at each trimester should be recommended for all pregnant women.		100% (Retained)		Retained	U
2.19.	Unselected routine serum ferritin measurement in the clinical setting is currently not recommended.	Serum ferritin measurement in women with anemia should be considered on a case-by-case basis.	63% (Included in Round 2)	75% (Non-consensus)	No consensus	C
2.20.	Serum ferritin may be used to monitor and assess the impact of interventions on iron status.		88% (Retained)		Retained	B
2.21.	In pregnant women, anemia in the first trimester is defined as an Hb concentration of less than 11.0 g/dL and can be stratified into 3 levels of severity: mild (Hb 10.0–10.9 g/dL); moderate (Hb 7.0–9.9 g/dL); and severe (Hb<7.0 g/dL).		100% (Retained)		Retained	U
2.22.	In pregnant women, anemia in the second trimester is defined as an Hb concentration of less than 10.5 g/dL and can be stratified into 3 levels of severity: mild (Hb 9.5–10.4 g/dL); moderate (Hb 6.5–9.4 g/dL); and severe (Hb<6.5 g/dL).		100% (Retained)		Retained	U
2.23.	In pregnant women, anemia in the third trimester is defined as an Hb concentration of less than 11.0 g/dL and can be stratified into 3 levels of severity: mild (Hb 10.0–10.9 g/dL); moderate (Hb 7.0–9.9 g/dL); and severe (Hb<7.0 g/dL).		100% (Retained)		Retained	U
2.24.	In pregnant women, anemia in the immediate postpartum period is defined as an Hb concentration of less than 10.0 g/dL and can be stratified into 3 levels of severity: mild (Hb 9.0–9.9 g/dL); moderate (Hb 6.0–8.9 g/dL); and severe (Hb<6.0 g/dL).		88% (Retained)		Retained	B

Statements on diagnosis and assessment of ID/IDA	Clarified statement	Round 1 rating	Round 2 rating	Result	Round 2 grading
2.25. In non-pregnant adult women, anemia is defined as an Hb concentration of less than 12.0 g/dL and can be stratified into 3 levels of severity: mild (Hb 11.0–11.9 g/dL); moderate (Hb 8.0–10.9 g/dL); and severe (<8.0 g/dL).		100% (Retained)		Retained	U
2.26. In adolescent girls, anemia is defined as an Hb concentration of less than 12.0 g/dL and can be stratified into 3 levels of severity: mild (Hb 11.0–11.9 g/dL); moderate (Hb 8.0–10.9 g/dL); and severe (<8.0 g/dL).		100% (Retained)		Retained	U
2.27. In perimenopausal women, anemia is defined as an Hb concentration of less than 12.0 g/dL and can be stratified into 3 levels of severity: mild (Hb 11.0–11.9 g/dL); moderate (Hb 8.0–10.9 g/dL); and severe (<8.0 g/dL).		100% (Retained)		Retained	U
2.28. Specific signs due to IDA are clinically useful in establishing a diagnosis of IDA when their presence is a change from normal in individual patients.	Specific signs due to ID (e.g., pallor of the skin, conjunctivae, and nail beds) are clinically useful in facilitating a diagnosis of IDA.	75% (Included in Round 2)	63% (Non-consensus)	No consensus	D
2.29. In iron-replete pregnant women with anemia, additional diagnostic tests are required to determine the alternate causes of anemia and establish the optimal course of therapy.		100% (Retained)		Retained	U
2.30. In iron-replete non-pregnant adult women with anemia, additional diagnostic tests are required to determine the alternate causes of anemia and establish the optimal course of therapy.		100% (Retained)		Retained	U
2.31. In iron-replete adolescent girls with anemia, additional diagnostic tests are required to determine the alternate causes of anemia and establish the optimal course of therapy.		100% (Retained)		Retained	U
2.32. In iron-replete perimenopausal women with anemia, additional diagnostic tests are required to determine the alternate causes of anemia and establish the optimal course of therapy.		100% (Retained)		Retained	U
2.33. Additional diagnostic tests are not required for pregnant women with confirmed IDA.	In selected cases of pregnant women with IDA, further diagnostic testing may be necessary to clarify the multiple etiologies and additional interventions required to treat IDA.	50% (Included in Round 2)	100% (Retained)	Retained	U

Statements on diagnosis and assessment of ID/IDA	Clarified statement	Round 1 rating	Round 2 rating	Result	Round 2 grading
2.34. Additional diagnostic tests are not required for non-pregnant adult women with confirmed IDA.	In selected cases of non-pregnant adult women with IDA, further diagnostic testing may be necessary to clarify the multiple etiologies and additional interventions required to treat IDA.	50% (Included in Round 2)	100% (Retained)	Retained	U
2.35. Additional diagnostic tests are not required for adolescent girls with confirmed IDA.	In selected cases of adolescent girls with IDA, further diagnostic testing may be necessary to clarify the multiple etiologies and additional interventions required to treat IDA.	50% (Included in Round 2)	100% (Retained)	Retained	U
2.36. Additional diagnostic tests are not required for perimenopausal women with confirmed IDA.	In selected cases of perimenopausal women with IDA, further diagnostic testing may be necessary to clarify the multiple etiologies and additional interventions required to treat IDA.	50% (Included in Round 2)	100% (Retained)	Retained	U
2.37. A trial of oral iron therapy may be used to diagnose IDA in anemic pregnant women to avoid delays in patient care.	A therapeutic trial with oral iron may be used to diagnose and treat IDA in anemic pregnant women to avoid delays in patient care.	75% (Included in Round 2)	63% (Non-consensus)	No consensus	D
			Retained (n)	28*	

*Two statements that did not meet consensus threshold – 2.14 & 2.15 – were removed and excluded from Delphi Round 2.

Table S3. Delphi results for statements relating to the prevention of ID/IDA in women.

Statements relating to the prevention of ID/IDA	Clarified statement	Round 1 rating	Round 2 rating	Result	Round 2 grading
3.1. Preventive iron supplementation should be recommended for all pregnant women living in settings where screening for ID/IDA is not adequate.		100% (Retained)		Retained	U
3.2. Preventive iron supplementation should be recommended for all non-pregnant adult women living in settings where screening for ID/IDA is not adequate.		100% (Retained)		Retained	U

Statements relating to the prevention of ID/IDA	Clarified statement	Round 1 rating	Round 2 rating	Result	Round 2 grading
3.3. Preventive iron supplementation should be recommended for all adolescent girls living in settings where screening for ID/IDA is not adequate.		100% (Retained)		Retained	U
3.4. Preventive iron supplementation should be recommended for all perimenopausal women living in settings where screening for ID/IDA is not adequate.		88% (Retained)		Retained	B
3.5. Preventive iron supplementation should be offered to all pregnant women, irrespective of anemia prevalence levels, to prevent ID/IDA.		88% (Retained)		Retained	B
3.6. Preventive iron supplementation should be offered to all non-pregnant adult women living in settings with high prevalence of anemia (≥40%) to prevent ID/IDA.		100% (Retained)		Retained	U
3.7. Preventive iron supplementation should be offered to all adolescent girls living in settings with high prevalence of anemia (≥40%) to prevent ID/IDA.		100% (Retained)		Retained	U
3.8. Preventive iron supplementation should be offered to all perimenopausal women living in settings with high prevalence of anemia (≥40%) to prevent ID/IDA.		100% (Retained)		Retained	U
3.9. Irrespective of anemia prevalence levels, all pregnant women should be offered a daily dose of 30–60 mg of oral elemental iron and 0.4 mg of folic acid throughout pregnancy to prevent ID/IDA and improve maternal and neonatal outcomes.		88% (Retained)		Retained	B
3.10. In settings with high prevalence of anemia (≥40%), all pregnant women should be offered an increased daily dose of 60 mg of oral elemental iron and 0.4 mg of folic acid throughout pregnancy to prevent ID/IDA and improve maternal and neonatal outcomes.		75% (Included in Round 2)	100% (Retained)	Retained	U
3.11. In settings with low prevalence of anemia (<20%) and if daily iron dose is poorly tolerated, all pregnant women should be offered an intermittent dose of 120 mg of oral elemental iron and 2.8 mg of folic acid once weekly to prevent ID/IDA and improve maternal and neonatal outcomes.	In settings with low prevalence of anemia (<20%), all pregnant women should be recommended weekly intermittent dose of 120 mg of oral elemental iron and 2.8 mg of folic acid to prevent ID/IDA and improve maternal and neonatal outcomes.	63% (Included in Round 2)	75% (Non-consensus)	No consensus	C
3.12. In settings where with gestational anemia is of public health concern, all postpartum women should be offered an oral iron supplement, either alone or in combination		100% (Retained)		Retained	U

Statements relating to the prevention of ID/IDA	Clarified statement	Round 1 rating	Round 2 rating	Result	Round 2 grading
with a folic acid supplement, for 6–12 weeks following delivery to prevent ID/IDA.					
3.13. In settings with high prevalence of anemia (≥40%), all non-pregnant adult women should be offered a daily dose of 30–60 mg of oral elemental iron for 3 consecutive months in a year to prevent ID/IDA.		75% (Included in Round 2)	88% (Retained)	Retained	B
3.14. In settings with high prevalence of anemia (≥40%), all non-pregnant adult women planning a pregnancy should be offered a daily dose of 30–60 mg of oral elemental iron and 0.4 mg of folic acid begun as early as possible and then continued throughout pregnancy and for 3 months postpartum to prevent ID/IDA and improve maternal and neonatal outcomes.		88% (Retained)		Retained	B
3.15. In settings with high prevalence of anemia (≥40%), all adolescent girls should be offered a daily dose of 30–60 mg of oral elemental iron for 3 consecutive months in a year to prevent ID/IDA.		88% (Retained)		Retained	B
3.16. In settings with high prevalence of anemia (≥40%), all perimenopausal women should be offered a daily dose of 30–60 mg of oral elemental iron for 3 consecutive months in a year to prevent ID/IDA.		75% (Included in Round 2)	88% (Retained)	Retained	B
			Retained (n)	15	

Table S4. Delphi results for statements relating to the treatment of ID/IDA in women.

Statements relating to the treatment of ID/IDA	Clarified statement	Round 1 rating*	Round 2 rating*	Result	Round 2 grading
4.1. The goal of therapeutic iron supplementation is to correct anemia and replenish iron stores.		100% (Retained)		Retained	U
4.2. Oral ferrous iron should be recommended as the first-line of therapy for pregnant women with uncomplicated ID/IDA without comorbidities.		88% (Retained)		Retained	B
4.3. Oral ferrous iron should be recommended as the first-line of therapy for non-pregnant adult women with uncomplicated ID/IDA without comorbidities.		88% (Retained)		Retained	B
4.4. Oral ferrous iron should be recommended as the first-line of therapy for adolescent girls with uncomplicated ID/IDA without comorbidities.		88% (Retained)		Retained	B

Statements relating to the treatment of ID/IDA	Clarified statement	Round 1 rating*	Round 2 rating*	Result	Round 2 grading
4.5. Oral ferrous iron should be recommended as the first-line of therapy for perimenopausal women with uncomplicated ID/IDA without comorbidities.		88% (Retained)		Retained	B
4.6. Pregnant women diagnosed with mild IDA during pregnancy should be offered a daily dose of 120 mg of oral elemental iron and 0.4 mg of folic acid until normalization of Hb levels (Hb 11.0 g/dL or higher) before resuming the standard daily dose of 30–60 mg of oral elemental iron and 0.4 mg of folic acid to prevent ID/IDA and improve maternal and neonatal outcomes.		75% (Included in Round 2)	88% (Retained)	Retained	B
4.7. Postpartum women with Hb less than 10.0 g/dL within 48 h of delivery, who are hemodynamically stable, asymptomatic or mildly symptomatic, should be offered a daily dose of 120 mg oral elemental iron and 0.4 mg of folic acid until normalization of Hb levels (Hb 11.0 g/dL or higher).		88% (Retained)		Retained	B
4.8. If oral iron is poorly tolerated, pregnant women with confirmed IDA should be offered intravenous iron from the second trimester onwards to normalize Hb levels and replenish iron stores.		100% (Retained)		Retained	U
4.9. If oral iron fails to increase Hb and iron levels, pregnant women with confirmed IDA should be offered intravenous iron from the second trimester onwards.		100% (Retained)		Retained	U
4.10. If rapid correction of Hb and iron levels is required, pregnant women with confirmed IDA should be offered intravenous iron from the second trimester onwards.		88% (Retained)		Retained	B
4.11. If oral iron is poorly tolerated, postpartum women with confirmed IDA should be offered intravenous iron.		100% (Retained)		Retained	U
4.12. If oral iron fails to increase Hb and iron levels, postpartum women with confirmed IDA should be offered intravenous iron.		100% (Retained)		Retained	U
4.13. If rapid correction of Hb and iron levels is required, postpartum women with confirmed IDA should be offered intravenous iron.		100% (Retained)		Retained	U
4.14. Non-pregnant adult women diagnosed with mild IDA should be offered a morning dose of 30–120 mg of oral elemental iron with ascorbic acid on alternate days until normalization of Hb levels (Hb 11.0 g/dL or higher) and then continued for a minimum 3 months to replenish iron stores.		100% (Retained)		Retained	U

Statements relating to the treatment of ID/IDA	Clarified statement	Round 1 rating*	Round 2 rating*	Result	Round 2 grading
4.15. Adolescent girls diagnosed with mild IDA should be offered a morning dose of 30–120 mg of oral elemental iron with ascorbic acid on alternate days until normalization of Hb levels (Hb 11.0 g/dL or higher) and then continued for a minimum 3 months to replenish iron stores.		100% (Retained)		Retained	U
4.16. Perimenopausal women diagnosed with mild IDA should be offered a morning dose of 30–120 mg of oral elemental iron with ascorbic acid on alternate days until normalization of Hb levels (Hb 11.0 g/dL or higher) and then continued for a minimum 3 months to replenish iron stores.		100% (Retained)		Retained	U
4.17. There is a lack of evidence guiding the optimal therapeutic dosing of oral iron in non-pregnant adult women with moderate-to-severe IDA.		63% (Included in Round 2)	88% (Retained)	Retained	B
4.18. There is a lack of evidence guiding the optimal therapeutic dosing of oral iron in adolescent girls with moderate-to-severe IDA.		63% (Included in Round 2)	75% (Non-consensus)	No consensus	C
4.19. There is a lack of evidence guiding the optimal therapeutic dosing of oral iron in perimenopausal women with moderate-to-severe IDA.		63% (Included in Round 2)	88% (Retained)	Retained	B
4.20. Non-pregnant adult women diagnosed with moderate-to-severe IDA, in whom rapid correction of Hb and iron levels are required, should be offered intravenous iron.		100% (Retained)		Retained	U
4.21. Adolescent girls diagnosed with moderate-to-severe IDA, in whom rapid correction of Hb and iron levels are required, should be offered intravenous iron.		100% (Retained)		Retained	U
4.22. Perimenopausal women diagnosed with moderate-to-severe IDA, in whom rapid correction of Hb and iron levels are required, should be offered intravenous iron.		88% (Retained)		Retained	B
4.23. Non-pregnant adult women diagnosed with mild-to-moderate IDA should be offered a daily morning dose of 30–120 mg of oral elemental iron with ascorbic acid until normalization of Hb levels (Hb 11.0 g/dL or higher) and then continued for a minimum 3 months to replenish iron stores.		New statement (Included in Round 2)	75% (Non-consensus)	No consensus	C
4.24. Adolescent girls diagnosed with mild-to-moderate IDA should be offered a daily morning dose of 30–120 mg of oral elemental iron with ascorbic acid until normalization of Hb levels (Hb 11.0 g/dL or higher) and then continued		New statement (Included in Round 2)	75% (Non-consensus)	No consensus	C

Statements relating to the treatment of ID/IDA	Clarified statement	Round 1 rating*	Round 2 rating*	Result	Round 2 grading
for a minimum 3 months to replenish iron stores.					
4.25. Perimenopausal women diagnosed with mild-to-moderate IDA should be offered a daily morning dose of 30–120 mg of oral elemental iron with ascorbic acid until normalization of Hb levels (Hb 11.0 g/dL or higher) and then continued for a minimum 3 months to replenish iron stores.		New statement (Included in Round 2)	75% (Non-consensus)	No consensus	C
4.26. There is a lack of evidence guiding the optimal therapeutic dosing of oral iron in non-pregnant adult women with severe IDA (Hb<8.0 g/dL).		New statement (Included in Round 2)	75% (Non-consensus)	No consensus	C
4.27. There is a lack of evidence guiding the optimal therapeutic dosing of oral iron in adolescent girls with severe IDA (Hb<8.0 g/dL).		New statement (Included in Round 2)	75% (Non-consensus)	No consensus	C
4.28. There is a lack of evidence guiding the optimal therapeutic dosing of oral iron in perimenopausal women with severe IDA (Hb<8.0 g/dL).		New statement (Included in Round 2)	75% (Non-consensus)	No consensus	C
4.29. Combining multiple micronutrients with oral iron is a rational treatment strategy for pregnant women with ID/IDA.		New statement (Included in Round 2)	88% (Retained)	Retained	B
4.30. Combining multiple micronutrients with oral iron is a rational treatment strategy for non-pregnant adult women with ID/IDA.		New statement (Included in Round 2)	88% (Retained)	Retained	B
4.31. Combining multiple micronutrients with oral iron is a rational treatment strategy for adolescent girls with ID/IDA.		New statement (Included in Round 2)	100% (Retained)	Retained	U
4.32. Combining multiple micronutrients with oral iron is a rational treatment strategy for perimenopausal women with ID/IDA.		New statement (Included in Round 2)	100% (Retained)	Retained	U
4.33. Pregnant women diagnosed with mild-to-moderate IDA should be offered a daily morning dose of 30–120 mg of oral elemental iron with ascorbic acid until normalization of Hb levels (Hb 11.0 g/dL or higher) and then continued throughout pregnancy to replenish iron stores.		New statement (Included in Round 2)	86% (Retained)	Retained	B
			Retained (n)	26	

Figure S1. Clinical pathway algorithm for the identification, and diagnosis and assessment of ID/IDA in women.

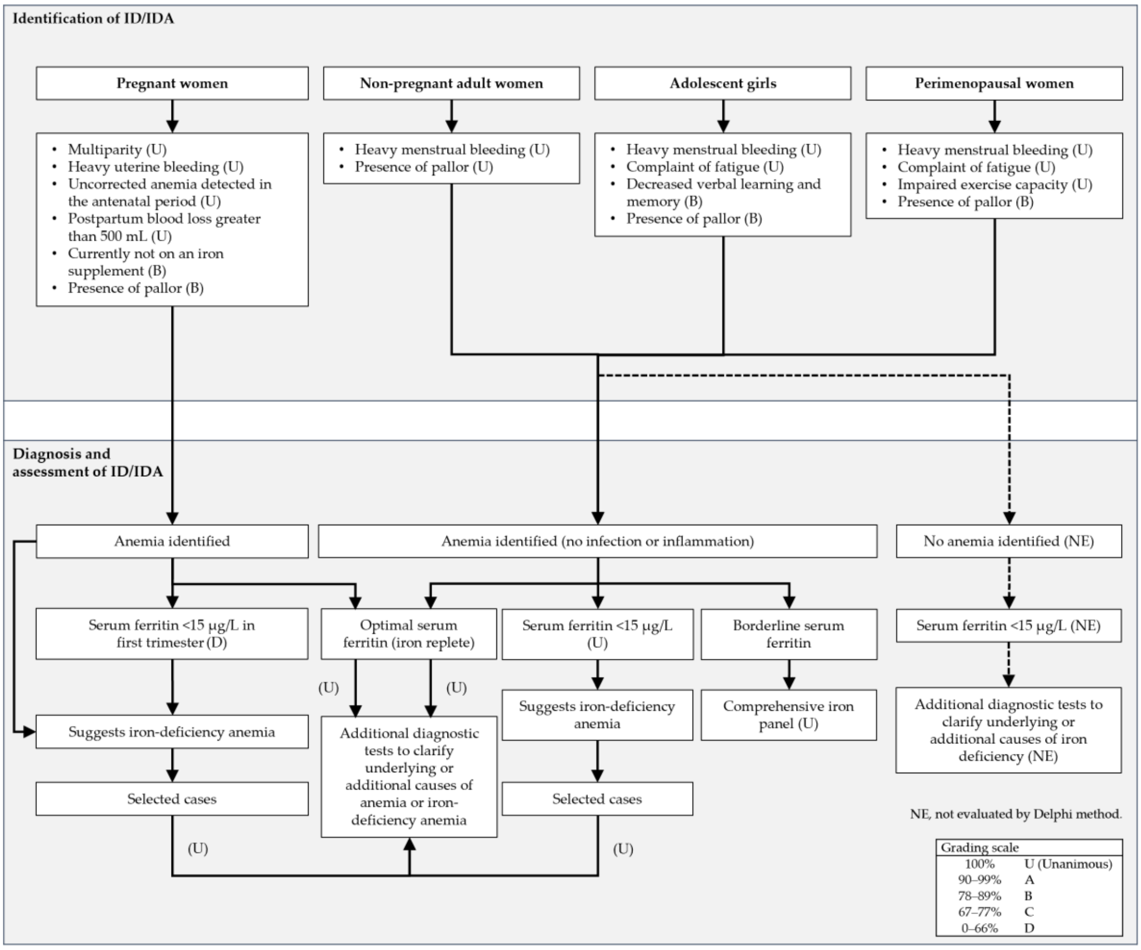

Figure S2. Clinical pathway algorithm for the prevention of ID/IDA in women.

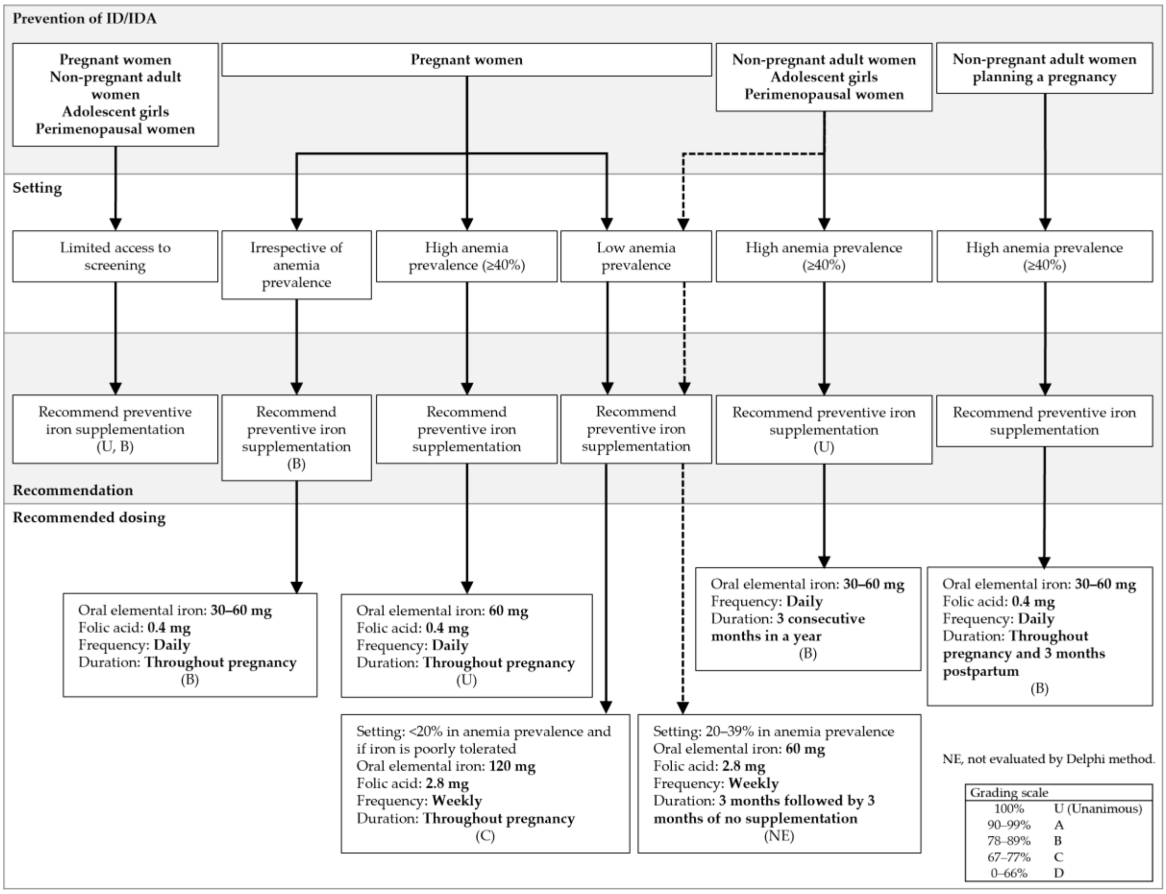

Figure S3. Clinical pathway algorithm for the treatment of ID/IDA in women.

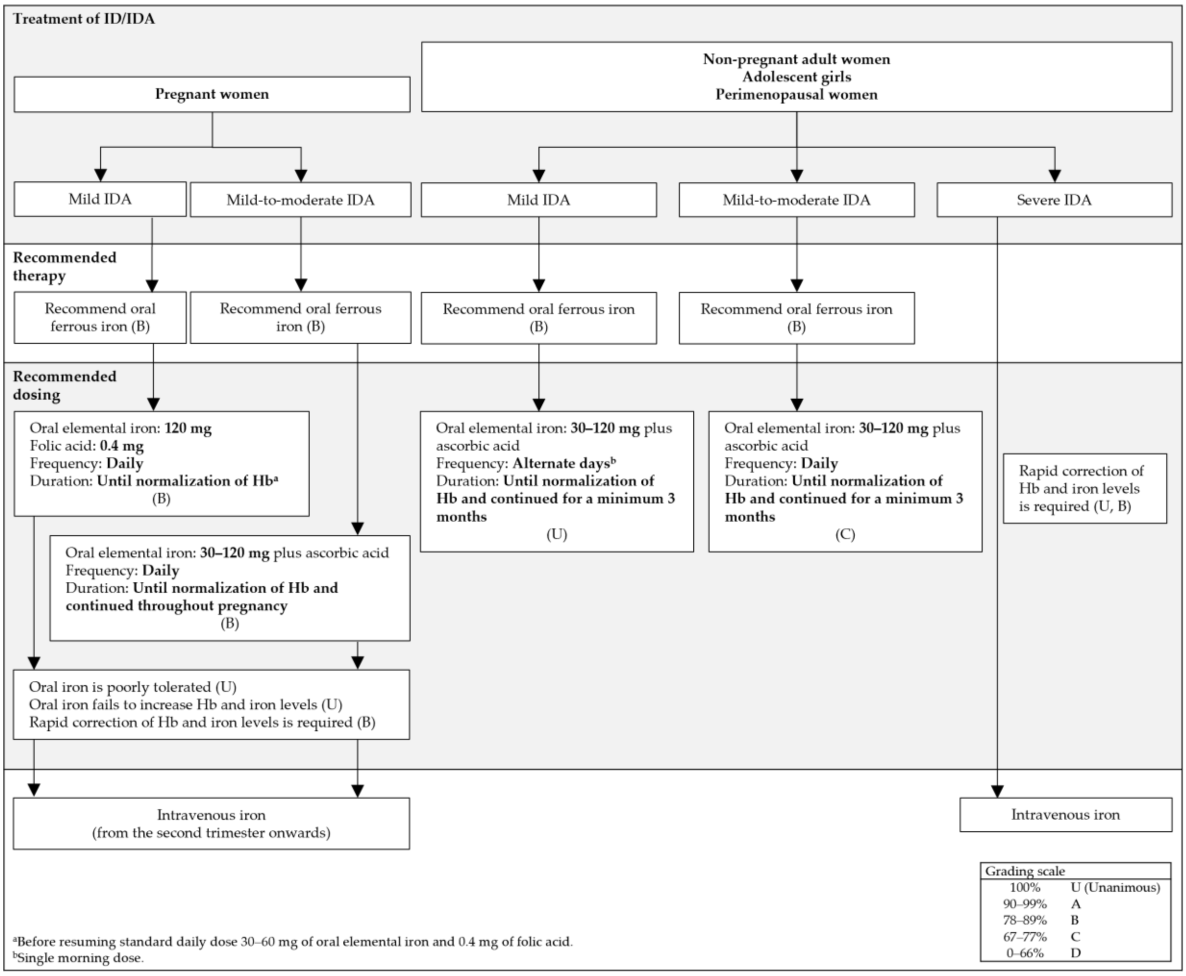

Supplement: Supplementary file 1 [file nutrients-15-03125-s001.zip › nutrients-2461164-supplementary.pdf]
